# “*False dichotomies*” and “*FND fundamentalists*”: an international mixed methods exploration of clinician and rheumatology patients’ views and experiences of functional neurological disorder (FND) co-diagnoses

**DOI:** 10.1007/s00296-026-06296-w

**Published:** 2026-10-16

**Authors:** Melanie Sloan, Guy Leschziner, Arjoon Arunasalam, David D’Cruz, Lucy Calderwood, Rupert Harwood, Arvind Kaul, Michael Bosley, Mervi Pitkanen, Sharmilee Gnanapavan, Max Yates, Alessandra Bortoluzzi, James A. Bourgeois

**Affiliations:** 1 Faculty of Medicine and Health, University of East Anglia, Norwich, UK; 2 Department of Public Health and Primary Care Unit, University of Cambridge, Cambridge, UK; 3 Department of Neurology, Guy’s and St Thomas’ Hospitals NHS Foundation Trust, London, UK; 4 Institute of Psychiatry, Psychology and Neuroscience, King’s College London, and South London and Maudsley NHS foundation trust, London, UK; 5 Department of Rheumatology, Guy’s and St Thomas’ Hospitals NHS Foundation Trust, London, UK; 6 Patient and Public Co-investigators, Cambridge, UK; 7 Swansea University Medical School, Swansea, Wales; 8 Department of Rheumatology, St George’s Hospital Trust, London, UK; 9 Department of Neurology, Bart’s Hospital Trust, London, UK; 10 Rheumatology Unit, Department of Medical Sciences, University of Ferrara and Azienda, Ospedaliero-Universitaria S. Anna, Ferrara, Italy; 11 Department of Psychiatry and Behavioral Sciences, Davis Medical Center, University of California, Sacramento, CA USA

**Keywords:** Rheumatology, Autoimmune, Functional neurological disorder, Mixed methods, Surveys and questionnaires, Clinician-patient communication

## Abstract

Functional neurological disorder (FND) presentations include limb weakness, tremors, non-epileptic seizures, and sensory symptoms. FND can present diagnostic challenges in systemic autoimmune rheumatic diseases (SARDs) due to overlapping neuropsychiatric symptoms. Evidence regarding its role and impact as a potential co-diagnosis in SARDs remains limited. Mixed methods data from international surveys and interviews were used to explore FND as a co-diagnosis in SARDs. Quantitative analyses included group comparisons using t-tests, ANOVA and Kruskal-Wallis tests as appropriate. Qualitative data were analysed thematically. Survey responses (N = 1853 patients, N = 400 clinicians, N = 463 general population) and in-depth interviews (N = 19 patients, N = 27 clinicians), indicated substantial variation in perspectives. Patients’ agreement with their co-diagnoses was higher for depression (86%) than for FND (30%). Participants described experiences suggestive of misattribution in both directions and perceived variation in diagnostic attribution between clinicians. Communication of an FND co-diagnosis varied; when interpreted as implying symptom controllability, this was associated with increased distress and reduced acceptance. Clinicians (72.1%) were significantly more likely than patients (50.2%) and the general population (47.9%) to state that mental health symptoms were considered more acceptable/less stigmatised if they were from the direct effects of the systemic disease (Welch’s F(2647) = 135.006), *p* < 0.01. Clinicians often used dualistic categorisations of symptoms (e.g., functional versus “organic”). Other clinicians considered dualism particularly unhelpful in SARDs due to the multiplicity, complexity and inter-relatedness of symptoms and aetiologies. FND as a co-diagnosis in SARDs is variably understood and applied, with differing levels of clinician agreement and patient acceptance. Clear, patient-centred explanations of diagnostic attribution, including how symptoms are interpreted and how symptoms being attributed to FND *differ* from SARD symptoms, may improve understanding and acceptance. These findings highlight the need for improved communication, multidisciplinary approaches and holistic care to support patients with complex neuropsychiatric symptoms.

**Supplementary Information:**

The online version contains supplementary material available at https://doi.org/10.1007/s00296-026-06296-w.

## Introduction

Systemic autoimmune rheumatic disease (SARDs) diagnosis and management can be complicated by co-morbidities. These can include additional autoimmune conditions, cardiovascular and metabolic disorders, psychiatric disorders, and fibromyalgia [1]. In addition, Joseph et al. report that 41.9% of their sample of functional neurological disorder (FND) diagnosed patients had co-morbid autoimmune diseases [2]. Differentiating symptoms is essential for diagnostic precision and planning optimal treatments, yet the commonality of symptoms and overlap among co-morbidities makes this differentiation challenging.

In DSM-5-TR [3], FND is recognised under the diagnosis of “conversion disorder (functional neurological symptom disorder)” and presentations include neurologic symptoms such as limb weakness, tremors, cognitive symptoms, non-epileptic seizures (seizures not caused by abnormal electrical activity in the brain) and sensory loss [4]. It is now framed as a “rule-in” diagnosis [5], with a shift toward diagnosis based on positive clinical signs of FND (e.g. Hoover’s test) rather than merely the exclusion of other explanatory illnesses. FND symptoms are now understood to be not consciously produced and there is no longer a diagnostic requirement of a specific psychological “trigger” associated with onset of symptoms. In DSM-5-TR, the diagnostic criteria include: “One or more symptoms of altered voluntary motor or sensory function”; “Clinical findings provide evidence of incompatibility between the symptom and recognized neurological or medical conditions”; and not “better explained by another medical or mental disorder” [3]. These latter two criteria may be particularly ambiguous in relation to SARDs patients. This is due to limited knowledge of the pathophysiological aetiology of diffuse relapsing-remitting neurological symptoms common in SARDs [6], and substantial overlap in presentation between SARDs-associated neurologic symptoms and FND neurologic symptoms.

FND is reported to be among the most common conditions seen in neurology outpatient clinics [7], and there is increasing diagnosis of FND with other chronic conditions. These includes Parkinson’s disease, long COVID and multiple sclerosis [8], although the literature is sparse on the co-occurrence of FND with rheumatological diseases. There may be a bidirectional relationship between autoimmune disorders and FND, as chronic systemic illness can lead to increased nervous system sensitisation, heightening vulnerability to functional symptoms in a reciprocal pattern [9]. Common co-diagnoses may be viewed with varying degrees of acceptability by both clinicians and patients. Attitudes can be influenced by limited knowledge and disagreement about causal mechanisms [6], and the greater stigma attached to diagnoses potentially having a “psychosomatic” component. The legacy of anachronistic terms such as “hysteria” and stigma persists. For instance, Annandale et al.’s review reports that clinicians “avoid, reject and dismiss patients with functional seizures and believe them to be ‘difficult’” [10]. Stigma and misunderstanding detrimentally impact access to health care and quality of life, and may affect whether a functional diagnosis is considered, communicated, and/or accepted by the patient [11].

Improved understanding of functional neurological disorder (FND) as a potential co-diagnosis in systemic autoimmune rheumatic diseases (SARDs), alongside more consistent diagnostic approaches, may have several potential benefits. These include supporting more appropriate alignment of treatment strategies with underlying mechanisms, broadening access to non-pharmacological interventions such as cognitive-behavioural therapies, and reducing the risk of diagnostic misattribution in both directions, including under-recognition of SARD-related neuropsychiatric symptoms and over-attribution to FND.

The aim of this study was to explore the acceptability, perceived validity, and clinical usefulness of FND as a co-diagnosis in people with SARDs from both patient and clinician perspectives.

## Methods

### Participants

Data were obtained from interviews and relevant sections of the international INSPIRE surveys [12] disseminated in 2022 online through charities, social media, and professional network recruiting via Qualtrics to: (1) People with any SARD as reported on clinical correspondence, and aged 18 or over, (2) People without a SARD or other autoimmune disease, and aged 18 or over, and (3) Clinicians of any speciality of registrar/resident level or above, and with current or previous experience with patients with SARDs. More detailed methods for the INSPIRE projects can be found in the Supplementary information of the first INSPIRE output [12].

### Study design and measures

Sequential mixed methods were used, with the quantitative data collected and presented first. The survey ascertained patient and clinician views as to whether common co-diagnoses were correct. For patients, this was phrased as to their level of agreement (using a Likert scale with 5 response options from ‘strongly disagree’ to ‘strongly agree’) that each stated co-diagnosis was correct in their own case. For clinicians, this was phrased as the estimated overall frequency of each diagnosis being *incorrect* in SLE with 7 response options ranging from ‘never’ to ‘always’. Likert scales were selected due to low burden and high accessibility for participants, and because this study sought to measure respondents’ perceptions and attitudes which are most appropriately captured through graded levels of agreement. Co-diagnoses options included a randomly ordered list of psychiatric (e.g. depression), somatic (e.g. anti-phospholipid syndome (APS)), “psychosomatic” and functional conditions. “Psychosomatic” was defined by our study psychiatrists as “physical symptoms caused or increased by psychological factors".

Qualitative data collection was through relevant responses to open-ended survey questions on views on co-diagnoses and in-depth interviews. Interviewees were purposively selected to ensure a range of sociodemographic characteristics and opinions on functional co-diagnoses. Guiding questions on co-diagnoses and FND included personal experiences and clinician communication in delivery of these diagnoses, views on attribution and treatment of symptoms, and patient-clinician interactions. Interviews were carried out using video conferencing or telephone by MS, a female academic with extensive interviewing experience. Interviews were audio-recorded and transcribed verbatim and continued until theoretical saturation was obtained.

### Analysis

Quantitative analysis and presentation are largely descriptive with percentages presented combining responses for ‘agree’ and ‘strongly agree’ into an overall agreement rating. Differences in mean values (using all 5 response items) between groups were tested for statistical significance using t-tests, ANOVA and Kruskal-Wallis tests as appropriate. Significance was pre-defined as an alpha of < 0.05. Due to attrition throughout the lengthy surveys and the ability to skip questions (for example, the clinician question on estimating misdiagnoses proportions), the denominator for each analysis is the number of participants completing that question.

Analysis of interviews and any relevant responses to open-ended survey responses was thematic using a constructionist approach. Following immersion and coding using NVivo12, themes were generated iteratively directly from the results. These were triangulated with the quantitative results, discussed, refined and agreed by the wider multidisciplinary team. Divergent findings and cases were considered and discussed.

### Patient partnership and monitoring

Patients were partners in every aspect of this study from study conceptualisation through design to analysis and write-up. The PIRIT tool and the GRIPP2 checklist (Table 1 supplementary) were used to monitor their influence and impact. The STROBE criteria were adhered to and can be found in supplementary (Table 2 supplementary). Diagnoses and misdiagnoses were reported by clinicians and patients and not independently verified; any conclusions are therefore related to interpretations of study participants’ experiences and perceptions of diagnostic accuracy/misattribution

## Results

Most patient participants were female (survey-91%, interviews-79%) (Table 1). The majority of clinician interviewees were rheumatologists (30%), neurologists (30%) and psychiatrists (26%). Four main themes were identified: (1) Disagreement regarding the accuracy and value of functional co-diagnoses; (2) Functional and/or relapsing-remitting symptoms misconstrued as “benign”; (3) Physician position on FND can influence diagnosis more than patient presentation; and (4) Communication of FND diagnosis shapes patient acceptance.

**Table 1:** Participant characteristics

Characteristic	Patient survey (n = 1853) (%)	Patient interviews (n = 19) (%)	Control survey (n = 463) (%)	Clinician survey (n = 400) (%)	Clinician interviews (n = 27) (%)
*Age*					
18–30	94 (5%)	3 (16%)	45 (10%)	16 (4%)	0 (0%)
30–39	195 (11%)	4 (21%)	71 (15%)	135 (34%)	7 (26%)
40–49	298 (16%)	3 (16%)	82 (18%)	135 (34%)	7 (26%)
50–59	519 (28%)	4 (21%)	84 (18%)	69 (17%)	6 (22%)
60–69 (60+ for clinicians)	478 (26%)	2 (11%)	120 (26%)	45 (11%)	7 (26%)
70+	267 (14%)	3 (16%)	60 (13%)	N/A	N/A
Prefer not to say	2 (<1%)	0 (0%)	1 (<1%)	0 (0%)	0 (0%)
*Gender*					
Female	1687 (91%)	15 (79%)	334 (72%)	209 (52%)	11 (41%)
Male	160 (9%)	4 (21%)	126 (27%)	186 (47%)	16 (59%)
Other/undisclosed	6 (<1%)	0 (0%)	3 (<1%)	5 (1%)	0 (0%)
*Country/region*					
England	1285 (69%)	6 (32%)	341 (74%)	156 (39%)	13 (48%)
Scotland	144 (8%)	4 (21%)	43 (9%)	16 (4%)	2 (7%)
Wales	104 (6%)	2 (11%)	20 (4%)	6 (2%)	3 (11%)
N. Ireland or Republic of Ireland	35 (2%)	0 (0%)	7 (2%)	2 (<1%)	0 (9%)
US or Canada	112 (6%)	2 (11%)	16 (3%)	65 (16%)	4 (15%)
Europe	121 (7%)	4 (21%)	24 (5%)	68 (17%)	2 (7%)
Asia	18 (1%)	1 (5%)	1 (<1%)	34 (9%)	1 (4%)
Latin America	4 (<1%)	0 (0%)	2 (<1%)	30 (8%)	2 (7%)
Australia or New Zealand	19 (1%)	0 (0%)	0 (0%)	10 (3%)	0 (0%)
Other	11 (<1%)	0 (0%)	9 (2%)	13 (3%)	0 (0%)
*Ethnicity*					
White	1718 (93%)	14 (74%)	434 (95%)		
Asian	49 (3%)	2 (11%)	6 (1%)		
Black	23 (1%)	1 (5%)	4 (1%)		
Mixed	40 (2%)	2 (11%)	11 (2%)		
Other/undisclosed	23 (1%)	0 (0%)	2 (<1%)		
*Disease*					
SLE	566 (31%)	6 (32%)			
Inflammatory arthritis	456 (25%)	3 (16%)			
Vasculitis	200 (11%)	2 (11%)			
Sjögren’s disease	150 (8%)	3 (16%)			
PMR	132 (7%)	0 (0%)			
UCTD	77 (4%)	2 (11%)			
Myositis	64 (4%)	2 (11%)			
Systemic sclerosis	63 (3%)	1 (5%)			
Mixed/multiple	145 (8%)	0 (0%)			
*Clinician role*					
Rheumatologist				204 (51%)	8 (30%)
Psychiatrist				96 (24%)	7 (26%)
Neurologist				52 (13%)	8 (30%)
Rheumatology nurse				20 (5%)	0 (0%)
GP/primary care				11 (3%)	3 (11%)
Other speciality				27 (7%)	1 (4%)

**Fig. 1:**
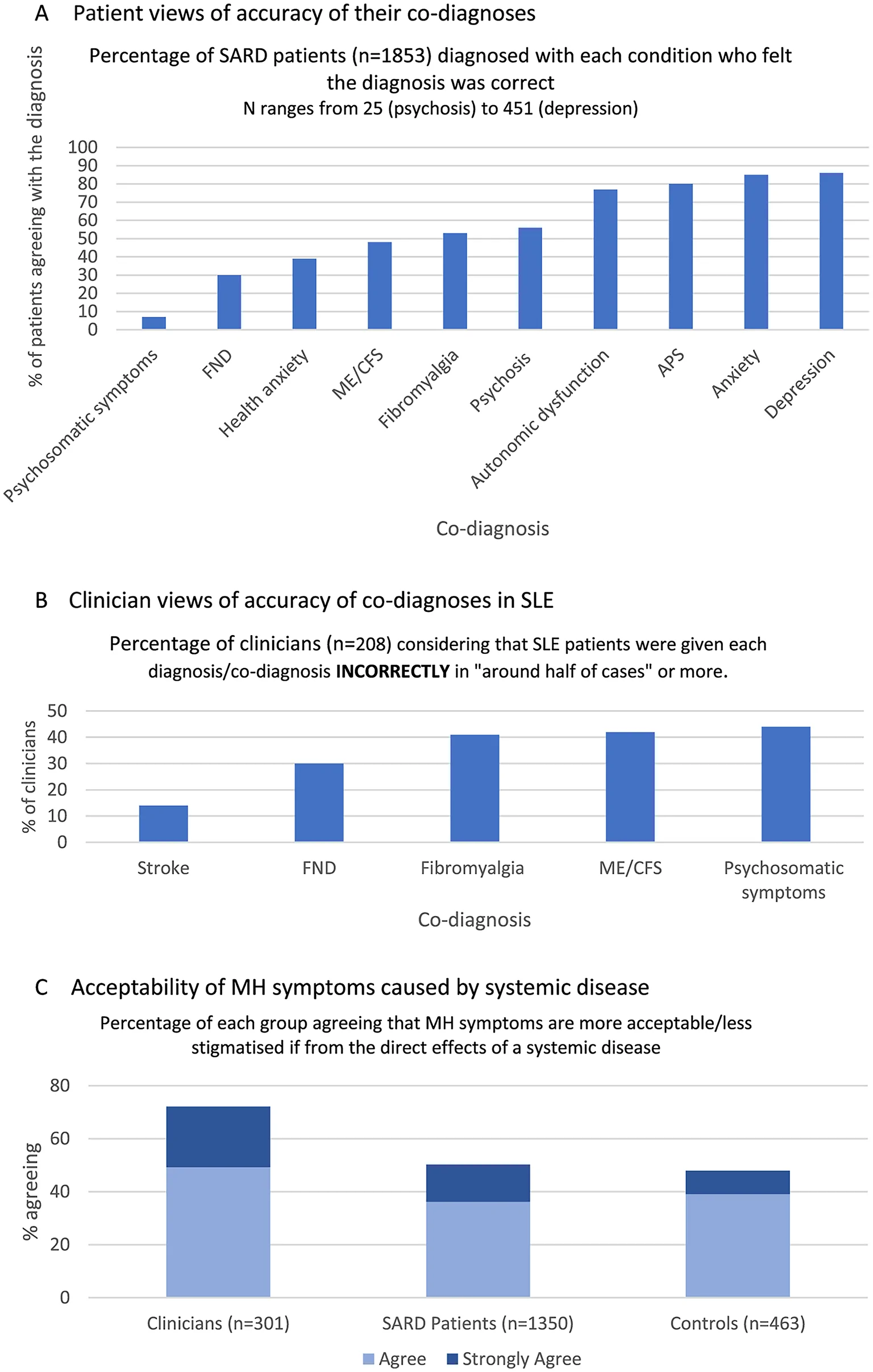
A–patient views of accuracy of their co-diagnoses. B–clinician views of accuracy of co-diagnoses in SLE. C–Acceptability of MH symptoms caused by systemic disease. Note: “Psychosomatic” was defined by our study psychiatrists as “physical symptoms caused or increased by psychological factors”. Abbreviations: FND = functional neurological disorder, APS = antiphospholipid syndrome, ME/CFS = myalgic encephalomyelitis/ chronic fatigue syndrome. Alt text – Bar graphs showing acceptability of co-diagnoses and proportion of clinicians (highest proportion), general population and controls considering MH symptoms to be more acceptable if from a systemic disease

Quantitative analysis demonstrated that patients’ agreement with the accuracy of co-diagnoses was much higher for depression (86%) than fibromyalgia (53%) or FND (30%), and lowest for diagnoses explicitly using the term “psychosomatic” (7%) (Fig. 1A). The proportion of clinician estimating that these co-diagnoses were incorrectly given in around half of all cases or more for lupus patients was 30% for FND, 41% for fibromyalgia and 42% for ME/CFS (Fig. 1B). Clinicians (72.1% of n = 301) were significantly more likely than patients (50.2% of n = 1350) and the general population (47.9% of n = 463) to state that mental health symptoms were considered more acceptable/less stigmatised if they were from the direct effects of the systemic disease (Welch’s F(2647) = 135.006), *p* < 0.01 when comparing the mean of the 5-item agreement rating for the 3 groups, and *p* < 0.001 when comparing patients (M = 2.84, SD = 1.131) with clinicians (M = 3.76, SD = 0.989), MD= −0.921, 95% CI [−1.053, −0.790], t(394) = −13.773) (Fig. 1C).

### Theme 1: disagreement regarding the accuracy and value of functional co-diagnoses

In interviews, there were strongly expressed conflicting views on functional co-diagnoses. Some clinicians strongly advocated for increased awareness and application of FND diagnoses, arguing how a co-morbid functional illness diagnosis facilitates more appropriate and targeted non-pharmacological treatment options for some symptoms. There were reports of inappropriate treatment from over-medicalising functional symptoms which may be more responsive to non-pharmacological measures:

*“They need to get them off all these medications, the overuse of opioids, it’s so wrong, people need counselling, they need support”* (Clinician 46, Neurologist)

Conversely, many patients reported symptom improvement, including of fatigue and cognitive dysfunction, when treated with corticosteroids and other immunosuppressants. Concern was expressed by clinicians and patients that functional and/or fibromyalgia illness labelling would reduce SARDs patients accessing immunosuppression that was reported by many as life-improving. Illustrative quotes of opposing opinions are reported in Table 2.

**Table 2:** Illustrative quotes summarising views of functional illness diagnoses

	Illustrative quotes in favour of considering *Indirect or functional* causes of symptoms	Illustrative quotes in favour of attribution of symptoms to the *direct effect* of autoimmunity
Overall	*For many patients and doctors the only narrative that allows that belief is if one attributes everything to the autoimmune condition itself…If it does turn out they do have co-morbid fibromyalgia, and having a disease like MS or lupus is a very strong risk factor for developing a co-morbid functional disorder, so do you really want to miss out on potential evidence-based treatment that can give you a better life and help with these co-morbidities because of these difficulties in talking about it and because of the stigma involved?* (Clinician 12, Neurologist)	*I think a lot of colleagues will dismiss fibromyalgia…It is the same with the neurologists and the functional neurology disorders, I think we as rheumatologists, we think these are pathological symptoms and look for the pathological cause, and how they are related to the* [rheumatologic] *disease activity. If there is no* [rheumatologic] *disease activity, only then will we dismiss symptoms as fibromyalgia or functional. I have seen many patients misdiagnosed with these in my career so maybe I am more wary* (Clinician 52, Rheumatologist)
Fatigue	*There are patients with the relapsing/ remitting form of fatigue more in line with the* [rheumatologic*] disease symptoms and clearly in those circumstances it’s directly disease related…*[but]*the vast majority will have fatigue where objectively and subjectively the* [rheumatologic] *disease isn’t always active…There are many strands that add to that fatigue and tiredness, and it’s where* [rheumatologic] *disease activity vs psychosocial* [attribution of causality*] is really planted heavily with lupus towards the psychosocial direction with fatigue* (Clinician 27, Rheumatologist)	*What goes on in autoimmune disorders is very fatiguing to the body, if you just think about energy metabolism, you know with mitochondria, so some antibodies have their own target, but there’s always that systemic response, because of all those inter-relations, it’s so fatiguing for the body to try and combat this. For the body to try and have homeostasis, or at least some degree, with that perpetual inflammatory response and inflammatory cascade, it just takes an enormous of energy to fight just that…So,* [regarding] *fatigue, I’ve definitely seen an enormous improvement on immunosuppression* (Clinician 58, Neurologist)
Sensory symptoms	*The waking up with tingling in one side of your hand and that’s just because your ulnar nerve got squashed in the night and it resolves quite quickly, we all get it, but once you’re worried, you’re just paying too much attention to these normal feelings….* [patients can] *become hypervigilant about their symptoms* (Clinician 66, Neurologist)	*I would say it definitely is part of the lupus…Generally you would have like “pins and needles”, you know, like if your foot goes dead or your arm goes dead, or something like that. People will get “pins and needles,” but it's not that kind of - it's not the same…It's a very bizarre feeling, but I would yeah, I would definitely say it's part of my lupus, I wouldn’t describe it as hypervigilance…* [it] *probably started less than a week prior to the psychotic episode.* (Patient 448, SLE)
Small fibre neuropathy (SFN)	*To say that the symptoms are neuro-lupus and need these very strong* [immunomodulation] *treatments is a risk. What is happening now, I’ve seen it with IVIG and people with sensory neurological symptoms seeing rheumatologists privately for IVIG. it’s just awful and they don’t need it, the damage caused and the damage of long-term* [cortico]*steroids.* (Clinician 41, Neurologist)	[Patient has] *been developing small fibre neuropathy and dysautonomia…never received confirmation of the diagnosis by several neurologists, until we referred her to a super-specialized neurologist. She was put on treatment with IV immunoglobulins with good results. She has enhanced symptoms in the 2 to 3 weeks prior to infusion* (Clinician 102, Rheumatologist)
Seizures	*I saw a patient with SLE who had functional seizures but attributed them to her lupus, and as a result absolutely refused to get off her immunosuppression…it took a very long time, about two years, but then she underwent some psychology* [psychotherapy] *and came off the immunosuppression, and the symptoms went away totally. It was absolutely clear that these symptoms weren’t due to the active inflammation* (Clinician 51, Psychiatrist)	*Problems with* [neurologists] *not understanding the disease or the nature of the disease or assuming you can always see things on an MRI scan... not recognising complex partial seizures which actually are pretty common in lupus. I’ve seen it and witnessed it in patients, and the neurologists try and say it’s not* [epilepsy], *and that it’s “functional” …They don’t always get it right, they don’t understand the nature of the disease or what it might respond to* (Clinician 2, Rheumatologist)

### Subtheme: localisation and the impact of (hypo/hyper/appropriate)vigilance

Although several neurologists highlighted the importance of localisation in direct attribution, others discussed that widespread “dysfunction” could also be explained by the biological effects of the disease:

*“It’s not about localisation; you often can’t localise these symptoms, but that doesn’t mean it’s ‘functional’ in the sense of that term. It’s usually explained by that inflammation and interaction; yes, it causes a lot of dysfunction, but that’s not what they mean with FND"* (Clinician 61, Neurologist)

Other clinicians indicated that many patients with SARDs and chronic pain will be likely unintentionally *“generating these symptoms as a result of that higher attention to your body”* (Clinician 58, neurologist), commonly described by clinicians as hypervigilance and/or interoception. Patients had wide-ranging views and experiences ranging from hypo- to hyper-vigilance (Table 3).

**Table 3:** Hyper-, hypo- or appropriate vigilance?

Hypovigilance	Combined hyper- and hypo-vigilance	Appropriate vigilance	Hypervigilance
Some patients described being conditioned by familiarity with multiple symptoms to not experience, or report, symptoms at the level someone who was experiencing them for the first time would: “*You just constantly downplay it all a lot, because when you start saying It out loud you think I can’t have all those symptoms, so you don’t say them all …, and when they say ‘how’s your pain?’, I might say ‘a 6’ but for a normal person they’d say ‘a 10. ’ We can cope with pain, because we’re used to it so we don’t look like ‘a 10’… there’s a normal scale and there’s an autoimmune scale”* (Patient 724, UCTD)	Other patients described a combination of both hypervigilance *and* hypovigilance: “*I was super aware of everything, right? Because it’s such a grey beast where it affects* [you]…*I can see how definitely you just become super self-conscious of anything in your body, [but] I extended [it] too much going into hospital, trying not to pay attention to it [so], I can see both sides…one is being super-aware thinking everything is a symptom, but also just thinking, well, this is normal because I always get this and then just ignoring it, there’s a bit of both”* (Patient 332, SLE)	Many patients felt that they were neither hyper- nor hypo-vigilant but exhibited a *necessary* level of vigilance, particularly given experience of symptoms not having been identified by clinicians and the need to closely monitor symptoms themselves: “*We aren't so much hypervigilant as justifiably attentive in managing a serious and complex chronic condition”* (Patient 910, Myositis)	Some patients identified with symptom amplification wherein they feel pain or sensations more strongly, potentially due to concurrent FND or fibromyalgia: “A *very simple touch on me, I can’t tolerate it, a simple touch can make me cry”* (Patient 191, SLE)

### Subtheme: untangling causation

When asked about differentiating between autoimmune and functional symptoms, the majority of clinicians acknowledged their limited knowledge and training: “*I never know*” (several clinicians), and “*I am not trained* [in this area]” (Clinician 217, GP). Several of the rheumatologists acknowledged very limited knowledge of FND, with one rheumatologist asking the interviewer: *“What’s a Hoover’s test?”* (Clinician 26, Rheumatologist)

Neurologists and psychiatrists were more familiar with the symptoms and signs of FND. Some highlighted the high prevalence of functional symptoms co-existing with autoimmune disease, yet also acknowledged the difficulty in differentiating causation: *“There is also a lot of functional neurology in lupus patients, and it is very hard to tease all the symptoms apart”* (Clinician 76, Neurologist), particularly with symptoms which have no objective markers or tests, such as fatigue and cognitive symptoms.

Functional seizures were unanimously accepted by clinicians as a valid diagnosis, yet attributing fatigue or cognitive dysfunction as functional was viewed with scepticism by many: “*I just cannot comprehend what the difference is between functional and non-functional fatigue, it just doesn’t make any sense*” (Clinician 51, Psychiatrist). Other clinicians felt that it was the direct effect of the disease on cognitive function: “*Many* [SLE] *patients may have subclinical neuroinflammation with long-term consequences of cognitive dysfunction”* (Clinician 189, Neurologist) (Table 4, sections 1 and 2)

**Table 4:** Identified problems and illustrative quotes

Problem(s) identified in interviews	Supporting quote(s)
**1. Limited knowledge and clinician disagreement**	
Lack of knowledge and teamwork	*“I do not feel qualified on my own to make this judgement”* (Clinician 219, Psychiatrist)
Poor inter-clinician consistency and conflicting views	“*Central nervous system involvement can be overlooked…I frequently see differences between several neurologists evaluating the same patient, which could suggest that it is not always easy to make a good neurological diagnosis in daily practice”* (Clinician 34, Rheumatologist*).* “*It’s so funny, the difference between us and neurologists, they see it as benign until it’s not”* (Clinician 52, Psychiatrist)
**2. Attribution challenges**	
Caution advised when both SARDs and FND cause similar symptoms	“*When a patient comes to clinic with a known ‘organic’ disorder like lupus, I think you should be very careful in diagnosing a functional disorder when we know that condition can involve the brain and cause these symptoms”* (Clinician 89, Neurologist). “*It is often possible with motor symptoms to be sure clinically that this is a functional symptom or a symptom due to structural damage, but doing this for subjective symptoms such as fatigue, pain and “cognitive fog” is much harder*” (Clinician 152, Neurologist)
Response to immunosuppression used as evidence of direct disease effect Most clinicians used response to immunosuppression and corticosteroids as evidence of inflammation. However, one patient self-attributed the improvement in her (diagnosed as non-epileptic) seizures following [cortico]steroids to the indirect effect of improved mood and reduced stress. One neurologist felt that perceived positive effects could often be attributed to placebo effect	“*We sometimes treat empirically and then are guided in diagnosis and management of it as neuropsychiatric lupus by their response to medication, especially the high-dose* [cortico]*steroids”* (Clinician 23, Rheumatologist) *“The seizures have stopped…I think when I’m on higher* [dose of cortico]*steroids my mood is better… it could be stress related, If I’m feeling negative and stressed it could bring on a seizure, but if I’m happy, then no seizure”* (Patient 724, UCTD). *“You can’t take much from patients’ reports and them saying they feel better on* [cortico]*steroids because the placebos can be very effective…But the ones we see who have been treated with rituximab and even cyclophosphamide, if their neurological symptoms haven’t got better that is evidence that this is purely a functional disorder”* (Clinician 66, Neurologist)
Response to talking therapy used as evidence of symptoms not being directly caused by the SARD A positive response of symptoms to psychotherapy was taken by some clinicians as evidence of the symptoms not being *directly* attributable to the SARD. However, some psychiatrists explained that psychotherapy and symptom-focused treatments can be equally as effective for some symptoms *directly* attributable (to SARDs), *indirectly* attributable, or *primary psychiatric disorder-related* psychiatric symptoms. Interviews also revealed that psychosocial support measures were valuable in reducing the impact of the symptoms on patients’ lives and ability to cope but often did not improve the symptom *itself*	*“Therapies helped me learn to pace myself while becoming resigned enough to my condition so I could calmly put up with all the miserable relentlessly progressing symptoms…but none of these therapies actually reduced the increase in intensity, persistence of the debilitation* [until immunosuppression was commenced]” (Patient 1,027, SLE)
**3. Insufficient support for all patients with life-changing diseases**	
Insufficient holistic care for patients	*“I was 18 when I got diagnosed, and that was a very hard thing for me to comprehend as a teenager. Therefore, I pushed it aside back then, and all of that stress and emotions about it, came back to haunt me years later”* (Patient 1361, SLE)
Towards applying the biopsychosocial model for all patients	*I think we need to have the biopsychosocial model for everyone…Understanding these things is important for treatment sometimes, but we need to get away from these distinctions and there’s so many other things, there’s the autonomic, there’s inflammatory and they all combine to impact on mood and the relationship with anxiety and everyone needs this whole holistic support, so we need psychologists and physiotherapists, too…. You are widening your treatment options, you might not find a cure, but you might benefit from targeting all different causes, so you might benefit [from]some drugs that slow heart rate, something that reduces histamine response, you might need to consider the psychological but improving the biological makes the biopsychology come together, if you know these things are all connected”* (Clinician 52, Psychiatrist)
**4. Poor diagnostic communication, distrust, and impact on future care**	
FND diagnoses often poorly delivered A method used to explain in a potentially more acceptable way to patients: “*If your body has experienced epileptic seizures, the way your brain works* [is] *that* [it] *can become the final pathway for the expression of any kind of physiological tumult that’s going on, so that’s why people with epileptic seizures then get functional seizures. Every kind of experience we have imprints on the brain…affect[s] the brain processing and functioning…in the same way if you’ve broken your leg, you may walk “funny” after. You’re at increased risk of having a “funny” walk even though it’s healed perfectly, because you’ve spent 3 months in a cast. Your brain has learnt new ways of walking, you may be scared of the consequences*” (Clinician 51, psychiatrist)	“*Making that narrative comprehensible and acceptable to the patient is something every doctor should do every time*…[yet] *sometimes, they are told, ‘you have FND, look on a website, not our problem, bye*’ “ (Clinician 41, Neurologist) *“I was not questioning him, I was just trying to explain what was happening inside my body and he said ‘What in the world are you saying, there is no such thing as what you are saying, it is not possible’. He said, ‘go and talk to somebody because you are mad’. He wasn’t even trying to understand me, he shouted, and I ran out into the lobby crying. I was just shattered by that. It was such a horrible experience*” (Patient 191, SLE)

### Theme 2: functional and/or relapsing-remitting symptoms misconstrued as “benign”

Symptoms were discussed as adversely impacted patients’ lives regardless of whether they were *directly related, indirectly related* or *unrelated* to the SARD, including those considered to be “benign” in pathology. In fact, participants reporting non-epileptic (functional) seizures often described more intense, prolonged and distressing episodes than those discussing their epileptic seizures, usually with less/no medical support.

Although several clinicians highlighted the importance of assisting patients in managing their non-directly attributable symptoms, this was rare, even though some symptoms were felt to be likely to respond better to non-pharmacological approaches. Some clinicians viewed the relapsing-remitting nature of many SARD NP symptoms as indicating that there would not be permanent physical damage. Others reported incidences of relapsing-remitting NP symptoms, in line with other rheumatologic disease activity, ultimately leading to permanent damage over time:

*“As they’ve got inflammation, you are going to be affecting the brain structures over time, so this is where we see end-organ brain damage for a lot of these neuroinflammatory disorders. It doesn’t show on scans and can remit a lot in the earlier stages, but you get to a point where you’ve lost a certain degree of *[function]*, when you go beyond the threshold, then it’s permanent. So, it can relapse-remit at first"* (Clinician 42, Neurologist).

This was re-iterated in accounts of patient experiences:

*"The phantom smells were not prioritized by my doctors… they called the* [multiple neurological symptoms] *"conversion disorder" *[until] *I had multiple confirmed strokes. I'm now permanently disabled"* (Patient 51, SLE).

Most clinicians also acknowledged the risk that an FND diagnosis could lead to stigma and misattribution of future SARD symptoms. This was apparent in patient experiences, including delayed diagnosis and treatment for SARDs, and potential diagnostic overshadowing from FND being on their medical records:

*"In A&E *[accident and emergency]* they said ‘Oh, you’re just in pain because of the lupus and it’s functional’… It’s almost like there’s a letter saying ‘hypochondria, there’s nothing wrong with her, put it down to fibro, put it down to functional neurological disorder, don’t take her seriously"* (Patient 724, UCTD).

### Theme 3: physician position on FND can influence diagnosis more than patient presentation

In interviews, clinicians often used terminology that implied symptoms fitted into dichotomous categories such as *organic* versus *functional*, *structural* versus *functional*, and *biological* versus *psychological*. Even the clinicians who expressed discomfort with these terms usually then used them, highlighting the difficulty that no suitable alternatives existed.

Analysis of clinicians’ opinions found three distinct groupings. The largest group (which comprised most of the rheumatologists) felt that not enough was known about the aetiology to have strong views. They advocated caution and being open-minded with each patient, acknowledging that “*there are many things that we do not know yet”* (Clinician 171, Neurologist) (Table 4, section 1 and 2) Neurologists were the most divided speciality, with one group suggesting that some colleagues were “*the worst dualists out there*” (Clinician 12, Neurologist) for classifying patients and symptoms according to rigid “*false dichotomie*s” (several neurology and psychiatry clinicians). This group felt that SARD patients were being over-treated with toxic [immunosuppressant] drugs, with one stating that this was “*negligence*” and *“cowardice”* (Clinician 41, Neurologist) in not addressing the psychiatric and social aspects of SARDs. An opposing group felt that some clinicians were too quick to attribute most symptoms to FND, hypervigilance and/or psychological causes and thus failed to consider the NP impact of systemic inflammation, with one psychiatrist labelling them “*FND fundamentalists*” (Clinician 202, Psychiatrist).

Interviews indicated firm divisions among clinicians on whether most NP symptoms were directly attributable to SARDs. For example, several neurologists stated that ‘most’ SLE patients will have FND, and that they knew before they even met them, or “*as they walked into clinic*” (Clinician 58, Neurologist), that the symptoms were not directly attributable to their disease. Others reported never diagnosing FND and keeping on searching until they found an alternative explanation:

*“As I work with patients with complex diseases, most of them have been given an FND diagnosis at some point by doctors who haven’t understood those systemic interactions of the immune and nervous systems. So, I spend time finding other explanations for their symptoms, which is almost always the case if you look in the right place” *(Clinician 61, Neurologist).

Several clinicians emphasised that functional disorders are no longer considered diagnoses of exclusion, and that there should be positive signs for diagnosis (such as Hoover’s test or distractible tremor for FND). However, other clinicians and many patients felt these diagnoses had been given due to negative test results. Testing was sometimes reported as either absent, cursory, inconsistent and/or subjective for FND and fibromyalgia: *“He pushed so hard *[that]* he left bruises…he said ‘the only thing wrong with you is fibromyalgia and hysteria”* (Patient 180, UCTD).

Certain physicians, departments and hospitals were reputed to more frequently give functional and/or fibromyalgia co-diagnoses than others. There were also occasional reports by clinicians and patients of the perception that some clinicians had research or personal agendas that they felt influenced the diagnoses: “*He* [The neurologist] *needed numbers for his research and he was just handing out FND labels here there and everywhere”* (Patient 1190, Sjögren’s disease).

### Subtheme: from false dichotomies towards holistic care

A common perspective was to endorse the integrated biopsychosocial approach: “*We can´t go on making a difference between body and mind”* (Clinician 91, Psychiatrist), particularly in terms of increasing the range of potential treatments beyond the purely pharmacological: *“To say which part is brain or body shouldn’t be relevant as they all connected”* (Clinician 52, psychiatrist). Several patients also felt their symptoms should be viewed holistically: *“You can’t actually say, ‘oh it’s physical or mental’… it’s all connected isn’t it”* (Patient 701, Sjögren’s disease).

Many clinicians and patients felt that it was also crucial to acknowledge the impact of the stress of having a chronic disease, both physically and emotionally. It was widely felt that most patients would also benefit from talking therapy and physiotherapy, whether their current symptoms were directly or indirectly attributable (Table 3, section 3).

### Subtheme: limited teamwork and trust

Most clinicians noted poor teamwork across specialties in SARD neuropsychiatric care, suggesting better multidisciplinary collaboration could resolve conflicts: “*I hope the outcome of your work is that people talk about it more and work together*” (Clinician 41, Neurologist). However, others were dismissive or distrusting of other speciality’s abilities in the area of attribution:

“*Neurologists tend to see things in black and white and that's not always appropriate with adisease like lupus, so I still send my patients to the neuroimmunologists, but I take what they say with a pinch of salt. I make my own mind up”* (Clinician 13, Nephrologist).

### Theme 4: communication of FND diagnosis shapes patient acceptance

There were many reports of functional diagnoses causing psychological distress, particularly the unempathetic delivery of co-diagnoses. This included patients perceiving a diagnosis as inferring “psychosomatic” causation without a contextual explanation that was understandable and acceptable to patients (Table 3, section 4). Repercussions included active suicidal ideation, suicide attempts, and one report of a patient citing the functional (mis)diagnosis in her suicide letter. Unempathetic communication of diagnosis(es) often generated shame and guilt from patients’ perceiving these diagnoses as implying that symptoms were within the patient’s control:

*“It’s that additional guilt then because they’re saying I could stop it* [non-epileptic seizures] *if I breathed better…The neurologist just said this is your diagnosis, just go and deal with it yourself… I had a lot of suicidal thoughts after that discussion, because I thought I can’t cope with this, if I’m doing this to myself, I need to end this, I need to end my life... I thought it was selfish of me because I was making him* [son] *suffer, and it was my fault”* (Patient 724, UCTD).

Several clinicians discussed having to either correct or clarify their colleagues inaccurate or poorly delivered co-diagnoses. Others discussed the importance of openness with patients when considering a potential psychological component and of including the patient views in any assessments. One patient compared the impact of an unempathetically delivered functional diagnosis with their later acceptance when a different clinician had empathetically communicated the rationale for co-diagnoses:

*“*[the endocrinologist] *explained* [FND] *as difference between ‘hardware and software’…*[whereas] *the neurologist basically said ‘this means there is nothing wrong with you and basically get over it and go away’”* (Patient 781, vasculitis).

In addition to explaining how symptoms differed between the systemic disease and co-diagnoses, listening, empathy and responsiveness assisted with acceptance and trust:

*“Oh my God, he’s* [neurologist] *amazing…He just listens, you know, we’d sort of like, we go properly into things…if I have something now that I don’t know whether it’s like the FND, or the Sjögren’s or what*[ever]*, so I just email him…and he’d email me back, like there and then”* (Patient 701, Sjögren’s disease)

Functional testing was reported as potentially reducing patient trust by being perceived as “*a bit underhand*” (Clinician 27, Rheumatologist) or to “*trick*” (several participants) the patient: “*They said it* [tremor] *was functional and I must be doing it to myself, because it stopped when they distracted me…But it comes and goes anyway... I said, “Why are you always trying to trick us?”* (Patient 1754, RA). Clinicians working in the FND field or psychiatry discussed the key importance of a biopsychosocial explanation but felt that many other clinicians could be dismissive of functional symptoms and that currently:

*“*[with FND] *there’s probably more bad experiences and bad* [clinician] *behaviour than good*” (Clinician 12, Neurologist).

## Discussion

We found a high level of strongly expressed disagreement regarding FND as a co-diagnosis in SARDs. Clinician participants described divergent clinical frameworks for interpreting neuropsychiatric symptoms, with some emphasising functional mechanisms and others prioritising direct immunological or neurological causes. Each side expressed concern that alternative approaches might lead to suboptimal care with perceived misdiagnoses reported in both directions. For patients with life-changing symptoms, extending the range of potential treatments to include non-pharmacological support should be of benefit. There is evidence of success of psychotherapy (e.g., CBT) and physiotherapy for FND, with one study reporting the success of structured psychotherapy giving patients “greater mastery over their stress responses” and therefore improving symptoms [13]. However, our findings suggest that, in practice, the introduction of an FND co-diagnosis may not consistently translate into access to these therapies, and may reduce access to immunosuppression for their inflammatory symptoms.

Although it has been asserted that misdiagnosis of FND is rare [14], more recent studies have found higher rates of underlying systemic medical conditions being misdiagnosed as FND [15]. Participants in our study reported perceptions of missed and misattributed diagnoses in both directions. A particular risk is the limited knowledge and multi-disciplinary teamwork, and clinician’s differences in their search for evidence either *for* or *against* a functional disorder diagnosis. Some clinicians described forming early diagnostic impressions, highlighting the potential influence of prior expectations on clinical reasoning. The literature also reports assumptions about patient behaviours that are reported as increasing suspicion of FND, including “attending alone” at the memory clinic [16] or the indoor use of sunglasses [17]. These findings suggest that clinician perspectives may contribute to variation in diagnostic attribution. This extends previous findings that patient sociodemographic characteristics can influence diagnosis more than presenting symptoms [18]. We observed substantial variability in viewpoints, and vast variations in the proportion (from ‘zero’ to ‘almost all’) of SARD patients that each clinician reported having/diagnosing with FND. Although positive signs such as the Hoover’s test [5] were viewed as objective and accurate by some clinicians, other clinicians and patients considered these tests (if used at all) to be subject to a high degree of perceived subjectivity and inter-clinician inconsistency.

The literature reports that clinicians can lack confidence in functional diagnoses, and biases can be apparent in the diagnosis delivery [19]. Communication of an FND diagnosis was reported as highly variable in our study, with the most favourable reports including clear explanations as detailed in the literature [20]. It was also found to be important to provide reassurance that symptoms were not the patient’s “fault” or “consciously controllable,” that it is a valid cause of symptoms like any other, and that the clinician would support recovery. Patient acceptance was influenced by explanations and agreement as to how any symptoms being attributed to FND *differed* from their SARD symptoms. Some participants described an adverse impact on their mental health from the perceived implication of controllability over these symptoms, and thus some blamed themselves, as found in other research [21]. In one case this had led to the functional misdiagnosis being cited in a suicide letter, and others detailed severe ongoing impact on their mental health and trust as highlighted in our previous study [22]. Moreover, participants reported that these co-diagnoses on medical records had sometimes adversely influenced other clinicians and caused diagnostic overshadowing and incorrect treatment. These findings align with the literature on diagnostic labelling and stigma [23]. Although it has been argued that FND stigma is on the decline due to greater “brain-based, mechanistic understanding of the condition” [24], stigmatised views were very apparent in many of our patient and clinician interviews.

The acknowledged limited awareness of FND by most rheumatologists in this study may contribute to under-identification of functional symptoms in SARDs. Conversely, knowledge of biological plausibility is continually evolving [25] which may influence how some diffuse neuropsychiatric symptoms in SARDs are interpreted in relation to the DSM-5-TR FND criteria of “not better explained by another medical or mental disorder” [3]. For example, small fibre neuropathy (SFN), increasingly understood to be common in SARDs [26], is one biologically plausible explanation for the frequent sensory symptoms that some clinicians view to be as a result of hypervigilance, and evidence for FND. Another frequent yet under-identified symptom of many SARDs is cardiovascular autonomic dysfunction which has been noted to be misinterpreted as “anxiety” in other diseases [27]. The converse is also true for functional symptoms mimicking symptoms requiring immunosuppression or antiseizure medication [28]. Although functional seizures can sometimes be differentiated from epileptic seizures by observations such as a longer duration or eyes being closed, some functional seizures are initially misdiagnosed as epilepsy [28]. These patients then rarely receive the psychiatric and other mental health care that can be effective, and the antiseizure medications will be ineffective and potentially dangerous.

One area of contention was regarding hypervigilance [29], described by some clinicians as a potential contributing factor to diffuse neurological symptoms due to excessive attention to normal sensations. However, patients with long-standing disease more frequently described having been conditioned by experiencing multiple symptoms to more commonly be *hypo-vigilant* rather than hypervigilant. In contrast to the hypervigilance theory, other clinicians suggested that many SARD patients will experience these neurological symptoms at higher frequencies and intensities due to the direct effect of the disease on the immune and nervous systems. Neurological symptoms may be more likely to be “psychologised,” as tests are usually “unenlightening” and symptoms are usually “invisible” to clinicians and are therefore more reliant on patient-reports [6]. Moreover, many patients have described in our broader INSPIRE programme of work how these neurological sensory and motor symptoms can be the first indication of an impending flare, including those necessitating organ and life-saving immunosuppression [30]. Patient vigilance to these symptoms of an impending disease flare may be required—and indeed encouraged—for earlier identification. Supporting patients to control their “hypervigilance”, and assuming it is maladaptive may be beneficial for some patients, but could have unintended consequences (for example, less prompt flare identification) if applied without consideration of disease context. In addition, our study raises concerns about the recommendation of some FND literature that clinicians should not reconsider a FND diagnosis once given. Given the similarity and overlap of neurological symptoms between SARDs and FND, and the reports of misdiagnoses, our findings support the importance of ongoing clinical review and diagnostic flexibility. This is particularly important in conditions characterised by fluctuating and overlapping symptom profiles.

## Strengths and limitations

Online, English-language only recruitment and the marked sex and ethnicity imbalance substantially limits representativeness and generalizability. Survey advertising was phrased neutrally and the content included multiple different topics in addition to the questions used for this paper so as not to skew respondents to those with strong opinions. However, there was nothing to prevent the survey link being shared with colleagues, thus potentially any incidental “snowball” recruiting may have been more likely to be with clinicians with shared opinions. Additionally, retrospective studies are subject to recall bias and all diagnoses and misdiagnoses were self-reported. Clinical records were not independently verified, and therefore reported misdiagnoses reflect participant perceptions rather than validated clinical determinations.

The qualitative findings should be interpreted as providing depth and context to the quantitative results rather than being statistically generalisable. A key strength of the study is the integration of large-scale international survey data with in-depth qualitative interviews, enabling triangulation across patient, clinician, and general population perspectives. The multidisciplinary design, combined with patient partnership throughout the research process, further strengthened interpretation and contextualisation of findings. This was particularly relevant during extensive discussions to ensure that all viewpoints - including those with lived experience -were considered and discussed when formulating themes from the data. The themes were wholly generated by the data as opposed to pre-conceived perceptions or influenced by the literature, thus reducing potential bias from external and internal influences.

## Conclusions

We found that FND is currently a variably understood and applied co-diagnosis with varying levels of acceptability among patients, and within and between specialisms. The challenges of attributing diffuse neuropsychiatric symptoms in SARDs highlight substantial clinical uncertainty in distinguishing between overlapping mechanisms. Simplistic dualistic conceptual frameworks, such as “functional versus structural” do not reflect the inherent complexity of many SARDs.

Although FND co-diagnoses may expand access to non-pharmacological treatment approaches and reduce unnecessary pharmacological treatment, participants also described potential risks, including diagnostic overshadowing, stigma, and under-treatment of the SARD. Improving patient-centred communication, clarifying the rationale for diagnostic attribution, and strengthening multidisciplinary collaboration may help to address these challenges. Whilst acknowledging resource limitations, *all* patients should be provided with appropriate disease management *and* offered access to supportive therapies tailored to their individual needs.

## Supplementary Information

Below is the link to the electronic supplementary material.

Supplementary file1 (DOCX 24 kb)

## Data Availability

Anonymised data will be available on reasonable request following the completion of the INSPIRE studies. Disclaimer: no part of this report is copied, published elsewhere (aside from related congress publication), or generated by AI. AI was not used for any aspect of this manuscript.

## Declarations

### Related congress publication

This work was presented at the British Society for Rheumatology Annual Conference 2026 and published in the conference proceedings in Rheumatology. DOI: keag121.084, https://doi.org/10.1093/rheumatology/keag121.084
