# Supplementary material for: “False dichotomies” and “FND fundamentalists”: an international mixed methods exploration of clinician and rheumatology patients’ views and experiences of functional neurological disorder (FND) co-diagnoses
Source: Rheumatol Int. 2026 Oct 16;46(11):297. doi: 10.1007/s00296-026-06296-w (PMC13714105; doi:10.1007/s00296-026-06296-w)
Supplement: Supplementary file 1 [file 296_2026_6296_MOESM1_ESM.docx]

**Supplementary information for: Differential attributions of neurological symptoms in rheumatologic diseases: An international mixed methods exploration of clinician and rheumatology patients’ views and experiences of functional neurological disorder (FND) co-diagnoses**

# GRIPP2-SF

Guidance for Reporting Involvement of Patients and the Public – short form^1^

| **Section and topic** | **Item** | **Reported in:** |
| --- | --- | --- |
| 1. **Aim**   *Report the aim of PPI in the study* | Patient and public partnership is a core ethos of the Long-Term Conditions Research Group. This includes partnering with patients at every stage of the research cycle including in priority setting. The aim is to help ensure that the research questions, design, analyses, findings and dissemination are fully reflective and representative of patient voices. | Methods |
| 2. **Methods**   *Provide a clear description of the methods used for PPI in the study* | Six patient and public partners contributed to this research in multiple ways as equal members of the wider research team including prioritisation of co-diagnoses. Roles included:   - Co-design and piloting of the survey questions. - Sharing and promoting the survey amongst patient communities and charities and social media.   Two patient partners were part of the core research team for this study and roles included:   - Co-designing and critically evaluating the semi structured interview guide. - Reviewing and discussing the literature and potential biases apparent in the literature and in care. - Reviewing relevant extracts of interview transcripts, advised on refining the coding framework and assisting in generating themes. - Attending and contributing to online, and F2F meetings with the full multi-disciplinary team and refinements via email. - Contributing sections to, reviewing and editing the manuscript and co-authoring this paper. | Summarised in methods |
| 3. **Study results**   *Report the results of PPI in the study, including both positive and negative outcomes* | This research overwhelmingly benefited from patient partnership, including at all stages listed in section 2 above. For example, benefits included ensuring that the survey and interview guide asked questions relevant to their own - and their peers - lived experiences, and ensuring questions were worded appropriately and that understanding was consistent between patients.  We gained unique perspectives from people with a range of diseases and ages.  Patient team members helped to interpret both the qualitative and quantitative data adding important perspectives of lived experience.  There were strong feelings about this topic and inclusion of a variety of specialities and lived experiences reduced possible clinician and researcher biases by ensuring discussions included the patient experience. | See results and strengths/limitations |
| 4. **Discussion and conclusions**   *Comment on the extent to which PPI influenced the study overall. Describe positive and negative effects* | Patient partners ensured that the focus of our research remained on the patient experience during every stage of the research cycle.  Partnering with patient partners had an extremely positive effect on the study. Positive effects were multiple and included greater depth of discussion on the long-term impact of feeling misdiagnosed and the importance of the diagnostic conversation.  The multidisciplinary design, combined with patient partnership throughout the research process, further strengthened interpretation and contextualisation of findings. This was particularly relevant during extensive discussions to ensure that all viewpoints - including those with lived experience - were considered and discussed when formulating themes from the data. | Discussion and strengths |
| 5. **Reflections/critical perspective**   *Comment critically on the study, reflecting on the things that went well and those that did not, so others can learn from this experience* | Whilst our long-standing working relationships with patient and public partners is a strength, our patient partners all have long-standing autoimmune disease and are well-educated adults. This may reduce the generalisability of their experiences to the newly diagnosed or those with broader socio-demographic characteristics.  Improvements we are working on include:   - Ensuring that the patient and public partners are more representative of the patient demographic, by inviting additional parters to the wider team. - Training and supporting interested patient partners to conduct some participant interviews by using peer interviewing. - Working with young people with SARDs to ascertain their views, experiences and priorities and partner with them in research. | N/A |

Reference

^1^ Staniszewska S, Brett J, Simera I, Seers K, Mockford C, Goodlad S, et al. GRIPP2 reporting checklists: tools to improve reporting of patient and public involvement in research. BMJ. 2017;358:j3453. DOI: <https://doi.org/10.1136/bmj.j3453>.)

**Table 2 – STROBE checklist**

| No | Content | Recommendation | Actioned |
| --- | --- | --- | --- |
| 1 | Title and abstract | (a) Indicate the study’s design with a commonly used term in the title or the abstract   (*b*) Provide in the abstract an informative and balanced summary of what was done and what was found | Yes, in the title  Yes, in the abstract |
| 2 | Introduction Background/rationale | Explain the scientific background and rationale for the investigation being reported | Yes, in the introduction |
| 3 | Objectives | State specific objectives, including any prespecified hypotheses | Yes, in the introduction |
| 4 | Methods – study design | Present key elements of study design early in the paper | Yes, in the main text and main INSPIRE supplementary material (referenced) |
| 5 | Methods - setting | Describe the setting, locations, and relevant dates, including periods of recruitment, exposure, follow-up, and data collection | Yes, in the main text and INSPIRE supplementary material |
| 6 | Participants | Give the eligibility criteria, and the sources and methods of selection of participants | Yes, in the main text and INSPIRE supplementary material |
| 7 | Variables | Clearly define all outcomes, exposures, predictors, potential confounders, and effect modifiers. Give diagnostic criteria, if applicable | Yes, in the main text |
| 8 | Data sources/ measurement | For each variable of interest, give sources of data and details of methods of assessment (measurement). Describe comparability of assessment methods if there is more than one group | Yes, in the main text |
| 9 | Bias | Describe any efforts to address potential sources of bias | Yes, in the main text and limitations |
| 10 | Study size | Explain how the study size was arrived at | Yes, in the main text |
| 11 | Quantitative variables | Explain how quantitative variables were handled in the analyses. If applicable, describe which groupings were chosen and why | Yes, in the main text |
| 12 | Statistical methods | (a) Describe all statistical methods, including those used to control for confounding (b) Describe any methods used to examine subgroups and interactions (c) Explain how missing data were addressed (d) If applicable, describe analytical methods taking account of sampling strategy (e) Describe any sensitivity analysis | In main text aside where applicable |
| 13 | Results | (a) Report numbers of individuals at each stage of study—eg numbers potentially eligible, examined for eligibility, confirmed eligible, included in the study, completing follow-up, and analysed (b) Give reasons for non-participation at each stage (c) Consider use of a flow diagram | Reported where relevant to this study |
| 14 | Descriptive data | (a) Give characteristics of study participants (eg demographic, clinical, social) and information on exposures and potential confounders | Yes, in Table 1 |
|  |  | (b) Indicate number of participants with missing data for each variable of interest | Yes, in the main text, graphs, and participant table |
| 15 | Outcome data | Report numbers of outcome events or summary measures | Yes, in main text |
| 16 | Main results | (a) Give unadjusted estimates and, if applicable, confounder-adjusted estimates and their precision (eg, 95% confidence interval). Make clear which confounders were adjusted for and why they were included (b) Report category boundaries when continuous variables were categorized (c) If relevant, consider translating estimates of relative risk into absolute risk for a meaningful time period | Study is largely descriptive |
| 17 | Other analyses | Report other analyses done—eg analyses of subgroups and interactions, and sensitivity analyses | N/A |
| 18 | Discussion  Key results | Summarise key results with reference to study objectives | Yes, in results section |
| 19 | Limitations | Discuss limitations of the study, taking into account sources of potential bias or imprecision. Discuss both direction and magnitude of any potential bias | Yes, in main text |
| 20 | Interpretations | Give a cautious overall interpretation of results considering objectives, limitations, multiplicity of analyses, results from similar studies, and other relevant evidence | Yes, in discussion, abstract and conclusion |
| 21 | Generalisability | Discuss the generalisability (external validity) of the study results | Yes, in discussion |
| 22 | Other information | Give the source of funding and the role of the funders for the present study and, if applicable, for the original study on which the present article is based | Yes, in main text |
